# Stereotactic Radiosurgery to the Nucleus Accumbens: A Dosimetric Feasibility Study

**DOI:** 10.7759/cureus.88231

**Published:** 2025-07-18

**Authors:** Hayden F Byrd, Guozhen Luo, Betsy Crosswhite, Godwin Emeka-Ibe, Taylor Davis, Ryan Whitaker

**Affiliations:** 1 Radiation Oncology, Vanderbilt University Medical Center, Nashville, USA; 2 Radiology, Vanderbilt University Medical Center, Nashville, USA

**Keywords:** addiction therapy, dosimetry analysis, linac-based srs, opium addiction, psychiatric neurosurgery, radiation for benign disease

## Abstract

Introduction: Addiction remains a critical issue in the United States, and options for individuals suffering from treatment-refractory addiction are limited. The nucleus accumbens (NAc) is a region of interest for both invasive and non-invasive interventions aimed at treating addiction. Stereotactic radiosurgery (SRS) is an established modality for managing benign conditions. This study seeks to establish the technical feasibility of targeting the NAc with linear accelerator-based SRS.

Methods: A single de-identified image set from a patient previously treated with linac-based SRS for a benign condition was used. Computed tomography images of the patient’s head, simulated in a stereotactic mask with corresponding high-resolution T1-weighted MRI brain and associated diffusion tensor imaging (DTI) data, were utilized for planning and localization. Three targets, named Ant, Mid, and Post, were placed on each side of the brain. Three separate plans were generated, targeting the bilateral NAc with one, two, and three shots to each side, with a maximal point dose of 140Gy, 120Gy, and 120Gy, respectively. The maximum dose to critical organs and volume of normal brain dose value V10Gy, V20Gy, and V60Gy were recorded.

Results: For a two-shot plan, the maximum point dose to bilateral anterior targets was set to be 140Gy. The maximum dose to the optic chiasm, optical nerves, and brainstem was 5.4Gy, 3.6Gy, and 3.5Gy, respectively, with brain V20Gy of 1.0cc and V10Gy of 3.8cc. For a four-shot plan, bilateral Ant and Mid targets were targets with a maximum dose set to 120Gy. The resulting maximum dose to optic chiasm, optical nerves, and brainstem was 9.0Gy, 5.2Gy, and 5.4Gy, respectively, with brain V20Gy of 1.9cc and V10Gy of 8.4cc. Similar plans were created for a six-shot plan, with a maximum point dose of 120Gy. However, the maximum dose to the optical chiasm, optical nerve, and brainstem were 17.1Gy, 5.2Gy, and 5.4Gy, andthe brain V20Gy and V10Gy were 4.4cc and 22.3cc, respectively.

Conclusion: We demonstrated the technical feasibility of targeting the NAc with SRS for the management of treatment-refractory addiction using a single sample patient. Two- and four-shot models appear to be most feasible to achieve the highest point-dose while respecting critical structure constraints. The attempt to add a six-shot drove the optical chiasm dose over our institutional constraint of 10 Gy in a single fraction due to the proximity to the chiasm. Demonstrating the efficacy and safety of radiosurgery to the NAc for addiction will require further carefully defined pre-clinical and clinical studies.

## Introduction

Substance abuse remains a critical issue in the United States, with approximately 13 million individuals receiving services dedicated to combating the illness in 2023, according to the National Survey of Drug Use and Health (NSDUH) [1]. Among the various forms of substance abuse disorders, those involving opioid analgesics have had a devastating impact on the United States populace, with over 80,000 reported preventable deaths in 2023, with over 700,000 since the year 2000 [1,2]. Financially, opioid-use-related disorders and fatal opioid overdoses were estimated to cost the United States $1.02 trillion in 2017 alone, with billions budgeted by the US government to combat the issue [1,3]. Great efforts have been made to improve care for individuals suffering from opioid abuse disorder; however, the disease course is challenged by cycles of relapse. Treatment options for these patients range from non-invasive cognitive therapies and pharmacological therapies to invasive interventions, including deep brain stimulation (DBS) and stereotactic ablative surgery [4-6]. Despite advances in understanding the pathophysiology and clinical treatments for opioid abuse disorder, new interventions are needed to assist in combating this morbid condition and similar diseases of abuse [7]. The nucleus accumbens (NAc) has been identified as playing a crucial role in neurological pathways associated with addiction [8,9]. Consequently, the NAc has become a region of interest for interventions aiming to treat addiction. Most of these treatments, such as stereotactic surgery and DBS, would require percutaneous intervention, which holds its own risk of morbidity and mortality [10]. Stereotactic radiosurgery (SRS) utilizes ionizing radiation to ablate tissues within the brain for both malignant and benign conditions, including treatment-refractory depression, obsessive-compulsive disorder (OCD), and essential tremor (ET), in a non-invasive manner [11]. To date, SRS to the NAc has not been studied. Before evaluating the safety and efficacy of SRS to the NAc for treatment-refractory addiction, preliminary data on the feasibility of targeting and delivering ablative doses of radiation to the NAc, while still meeting constraints to organs at risk (OARs), are required. This study sought to establish the technical feasibility of targeting the NAc with linear accelerator-based SRS.

## Materials and methods

**Figure 1:**
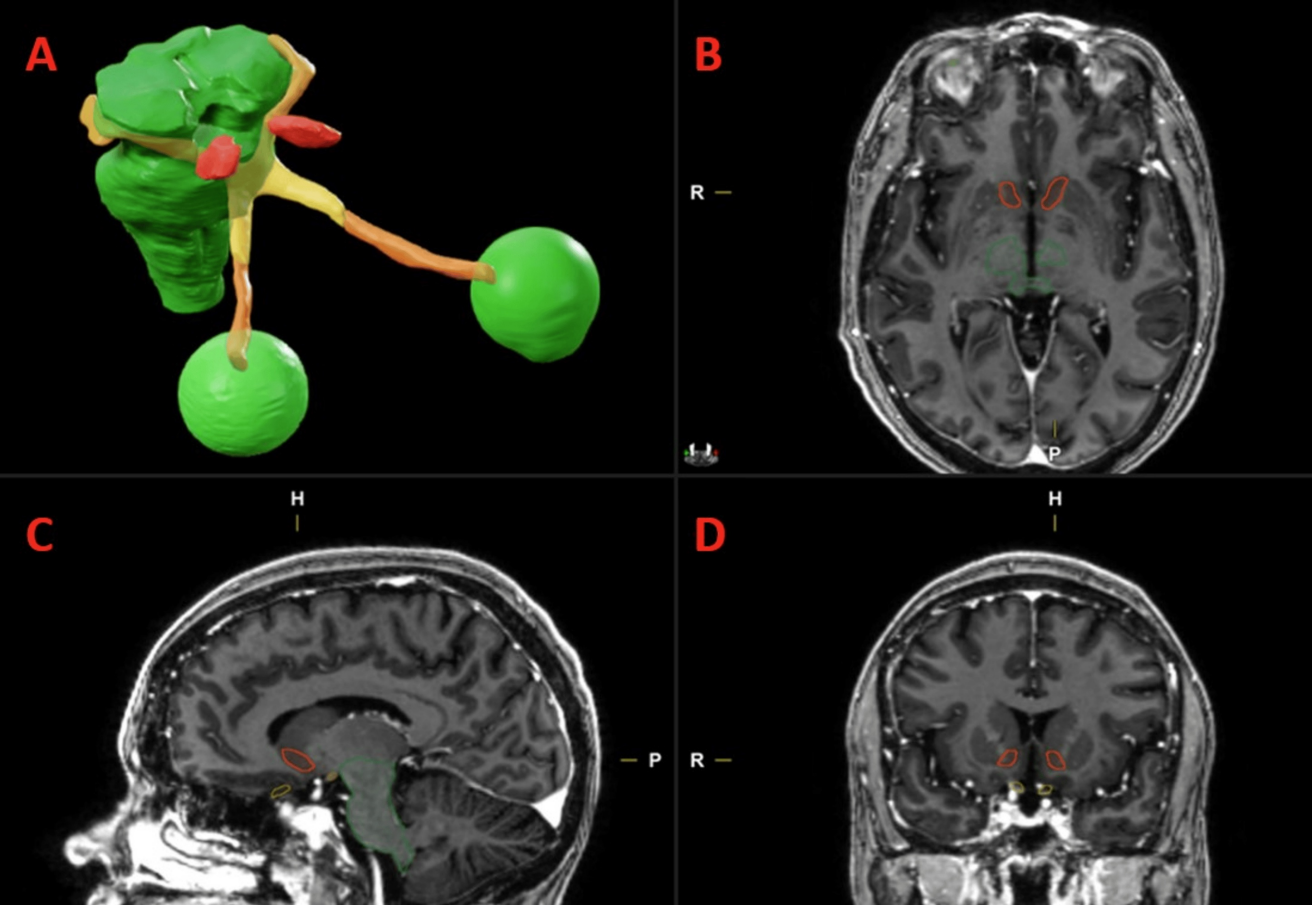
Critical structures Volumetric representations of brainstem (green); optic nerves, chiasm, tract (yellow/orange); eyes (green); and nucleus accumbens (red) (A). Volumes displayed on test patient MRI in axial (B), sagittal (C), and coronal (D) views.

**Figure 2:**
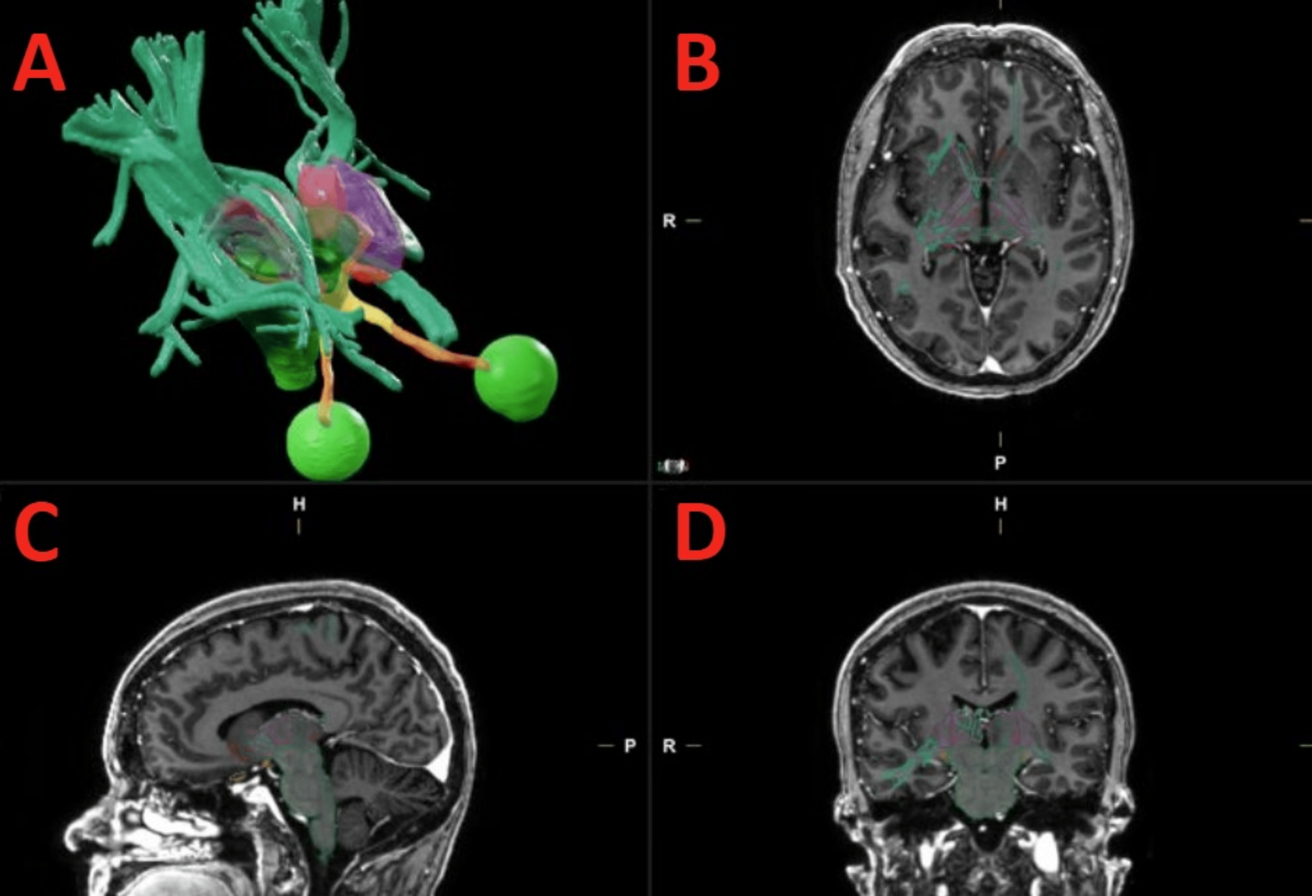
Additional critical structures Volumetric representative of the internal capsule (purple); thalamus (pink); and autosegmented radiations to and from the NAc based on DTI (teal) (A). Volumes displayed on test patient MRI in axial (B), sagittal (C), and coronal (D) views.

This is a single-institution dosimetric analysis performed at Vanderbilt University Medical Center. Following IRB approval, we utilized a single de-identified image set within the Brainlab treatment planning system, iPlanDose (version 4.5.5), from a patient previously treated at our institution with SRS for a functional disorder. The image set utilized included a computed tomography (CT) series of the patient’s head, which was simulated while positioned supine in a thermoplastic stereotactic mask. A corresponding T2-weighted MRI brain dataset and associated diffusion tensor imaging (DTI) data were utilized for planning and localization of the target and surrounding critical structures. The NAc was automatically segmented with the Brainlab Elements subthalamic contouring module, which is based on strong field MRI perfusion data, and then approved by a radiation oncologist and a neuro-radiologist referencing patient DTI data and established stereotactic and imaging databases as an independent check. Additional critical structures identified include eyes, optic tract, optic chiasm, optic nerves, brainstem, internal capsule, and thalamus (Figure 1, Figure 2).

**Figure 3:**
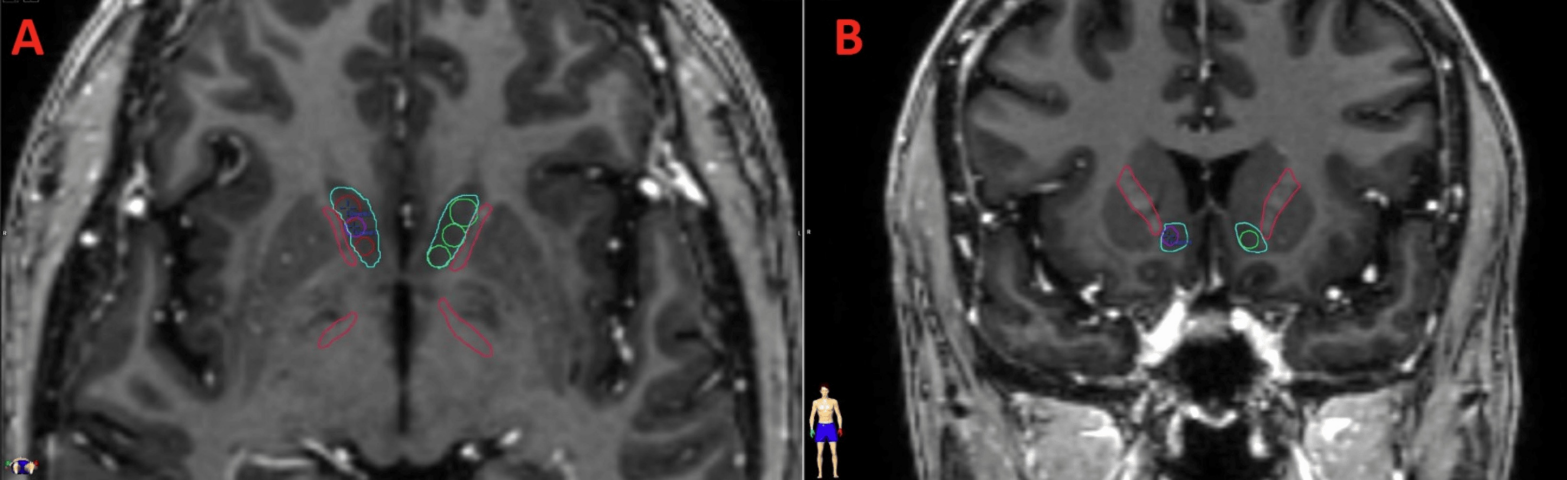
Ant, Mid, and Post trajectories displayed in the axial and coronal views Axial (A) and coronal (B) views of shot loci displayed as circles within nucleus accumbens (NAc) (teal) with adjacent internal capsule (pink).

Three targets (Ant, Mid, Post) were placed on each side of the brain (Table 1, Figure 3) for six shots total. Isocenter coordinates were decided based on the anatomy of the patient. Circular targets with 4mm diameter centered around the isocenters were manually placed. Utilizing an in-house plan template, one plan for each isocenter coordinate was created. Three separate plans were generated, targeting the bilateral NAc with one, two, and three shots with doses of 140Gy, 120Gy, and 120Gy, respectively. All the shots utilized a 4mm conical cone. For each shot, 15 arcs were utilized. With Varian convention, for the right side shots, couch angles were 260°, 250°, 240°, 230°, 220°, 180°, 172°, 162°, 152°, 142°, 132°, 122°, 112°, 102°, and 92°. Couch angles for the left side shots mirrored those of the right side shots. The total gantry angle was 100° for each arc. The maximum doses to critical organs were reported in Gy. Plans were renormalized for the maximum dose of each target equal to the prescription dose. As the targets abut each other on each side, the region of abutment is where the maximum dose is located. The volume of brain receiving at least a specified dose (V10Gy, V20Gy, and V60Gy) was recorded in cubic centimeters. Given that the closest distance of the optic chiasm to the six target planes was less than 10mm, we did not set the optic chiasm as an avoidance structure.

**Table 1:** Trajectories for each shot based on the axis centered on the corpus collosum

Trajectory No.	X	Y	Z
Trajectory 1: L Post	5.19	70.63	245.31
Trajectory 2: L Mid	8.19	66.64	245.55
Trajectory 3: L Ant	10.01	62.25	247.35
Trajectory 4: R Post	-9.80	68.70	246.19
Trajectory 5: R Mid	-10.79	64.97	247.28
Trajectory 6: R Ant	-11.77	61.58	248.95

## Results

**Figure 4:**
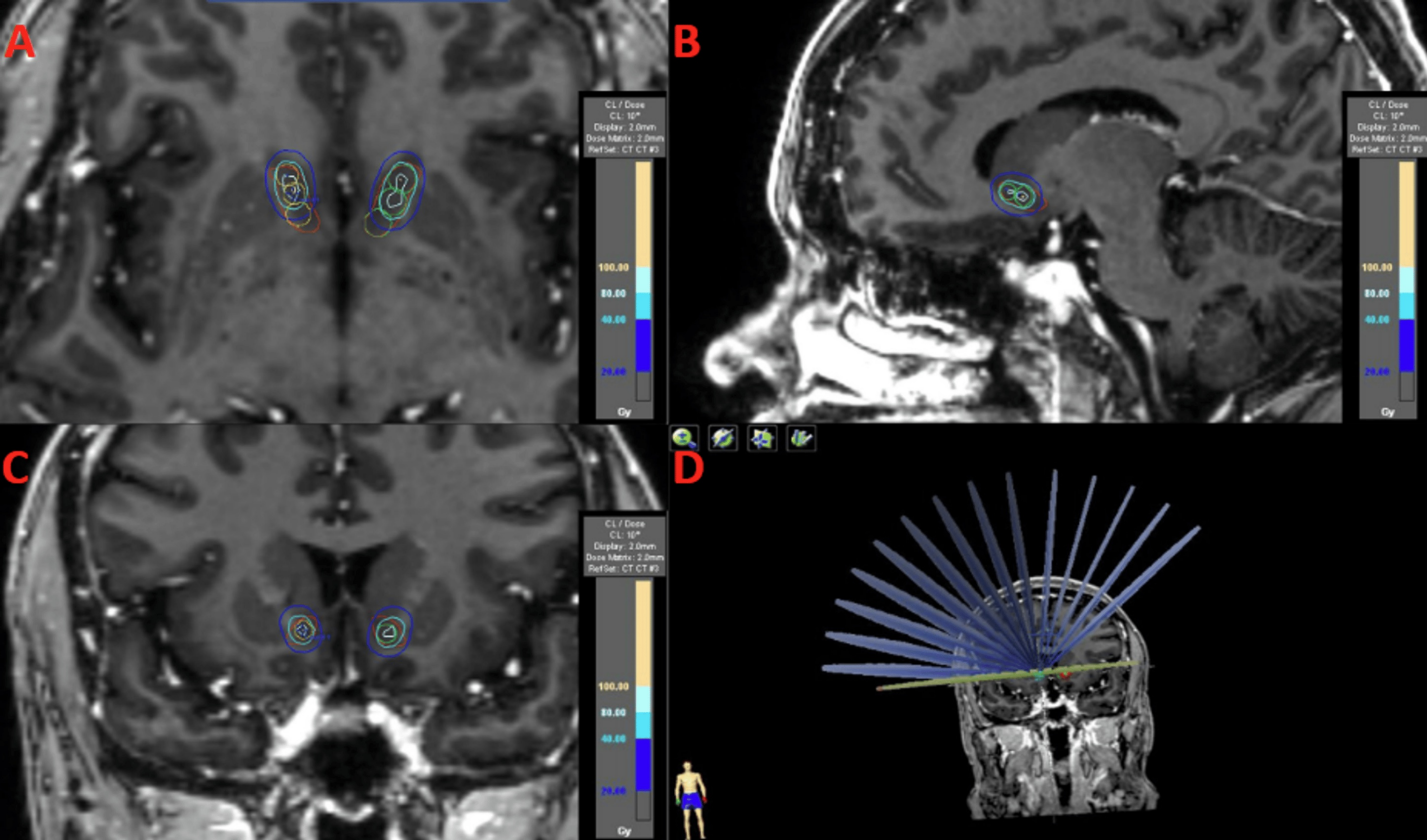
Dosimetry of the six-shot plan Six-shot plan displayed in axial (A), sagittal (B), and coronal (C) views with the corresponding dose reference and treatment arcs (D).

Overall, 19 plans were generated over an iterative process with combinations of shot location and balancing arc weighting to determine the three most optimal plans based on the target goal and OAR constraints. For the optimal two-shot plan, the maximum point dose to bilateral anterior targets was set to be 140Gy. The maximum dose to the optic chiasm, optical nerves, and brainstem was 5.4Gy, 3.6Gy, and 3.5Gy, respectively, with brain V20Gy of 1.0cc and V10Gy of 3.8cc (Table 2, Table 3). For a four-shot plan, bilateral Ant and Mid targets were targets with a maximum dose set to 120Gy. The resulting maximum dose to optic chiasm, optical nerves, and brainstem was 9.0Gy, 5.2Gy, and 5.4Gy, respectively, with brain V20Gy of 1.9cc and V10Gy of 8.4cc. A similar plan was created for a six-shot plan with a maximum point dose of 120Gy (Figure 4). However, the maximum dose to the optical chiasm, optical nerve, and brainstem was 17.1Gy, 5.2Gy, and 5.4Gy, and brain V20Gy and V10Gy were 4.4cc and 22.3cc, respectively.

**Table 2:** Institutional constraints utilized Maximum dose (Dmax) in gray. Dmax ≤ 150% denotes that target receives less than or equal to 150% of the prescribed dose. V100% > 97% meaning greater than 97% of the target volume receiving 100% of the prescribed dose.

Critical structure	Target Dmax	Optic chiasm	Optic nerve	Brainstem
Institutional constraint	Dmax ≤ 150%; V100% > 97%	Dmax 10Gy	Dmax 10Gy	Dmax 15Gy

**Table 3:** Plan outcomes for two-, four-, and six-shot plans Maximum dose reported in gray and volumetric dose reported in cubic centimeters (cc).

Shots	Target Dmax	Optic chiasm	Optic nerve	Brainstem	Brain V10Gy	Brain V20Gy	Brain V40Gy	Brain V60Gy
Two shot	140Gy	5.4Gy	3.63Gy	3.54Gy	3.8cc	1.0cc	0.32cc	0.19cc
Four shot	140Gy	9.0Gy	5.24Gy	5.37Gy	8.4cc	1.9cc	0.70cc	0.26cc
Six shot	120Gy	17.11Gy	5.24Gy	5.37Gy	22.3cc	4.4cc	0.96cc	0.45cc

## Discussion

Drug addiction arises from an interplay of social, behavioral, and neurobiological factors. The NAc is believed to play a pivotal role in the mechanisms of drug addiction [8]. Located within the ventral striatum, it acts within the mesolimbic system, receiving dopaminergic projections from the ventral tegmental area (VTA) in the brainstem and thereby propagating pathways responsible for pleasure, including those brought about by substances such as alcohol or opioids [12-14]. Because of this, the NAc is a logical target for interventions seeking to disrupt the pleasure-seeking cycles that characterize addiction. Prior surgical studies have evaluated the utilization of stereotactic ablation of the NAc [5,15]. While the ethical considerations of recruitment, consent, and treatment of individuals with substance abuse pose challenging obstacles, initial data on the utility of surgical intervention showed promising efficacy with regard to opiate abstinence. Wang et al. [15] observed that inclusion of the bilateral anterior and lateral aspects of the NAc, as well as the shell and core, yielded abstinence levels at around 75% at four years. In comparison, groups where only the shell or the majority of the focus was extending beyond the NAc were ablated, producing abstinence rates of approximately 30%. The greatest nonrelapse group included six foci, suggesting that the expanded field contributes to abstinence rates [5,15]. While this did not come at the cost of statistically higher incidence of anterograde amnesia, anhedonia, or affective disorders compared to the groups that received more medial and posterior foci, the reported 24% incidence of neuropsychiatric adverse events is worth noting [15].

The benefit of SRS in this setting is its non-invasive nature, compared to percutaneous interventions, which contain the inherent risk of infection and bleeding, potentially leading to highly morbid outcomes. With the addition of the emerging capabilities in tractography, we sought to establish the ability of SRS to accurately target the NAc. Advances in autosegmentation software with Brainlab Elements allowed for delineation of the NAc, which we verified by pairing the sample patient’s DTI data. Following review by a neuroradiologist, the autosegmented structures were modified slightly for better delineation of the internal capsule/NAc interface. Based on prior experiences treating benign neurological conditions such as OCD and depression, we believe that it is necessary to deliver a high dose in the 120-140Gy range, keeping in mind the neurosurgical data that treating the anterolateral aspect of the NAc was associated with the highest levels of long-term abstinence [11]. That being said, the authors acknowledge animal and human data, which suggest that lower doses, including down to 90Gy, may be sufficient to provide a neuromodulatory effect [16,17]. In addition, the aforementioned neurosurgical study had the best outcomes when at least four foci encompassing the shell and core bilaterally were ablated [15]. We were able to achieve a maximum dose of 140Gy with the two and four-shot plans. We attempted to increase to a six-shot plan; however, this was not feasible as we exceeded our institutional single fraction constraint to the chiasm of 10Gy. The closest distance of the optic chiasm to the six target plans was less than 10mm. Therefore, if the optic chiasm were set as an avoidance structure to potentially decrease dose to within acceptable limits, arc lengths would have to be significantly shortened. The result of that would be that there would not be enough arc degrees to ensure enough dose fall off to the normal brain, and brain V20 would be significantly higher.

This study was limited in its scope as a pre-clinical, technical feasibility study to suggest a framework for further investigation. Other treatment planning systems and radiosurgery systems, such as IMRT with microMLCs or Gamma Knife ®, were not considered; however, we believe that this should be technically achievable across modern radiotherapy platforms. Additionally, we were limited in our beam energy selection to a 6MV beam when planning. Lower energy beams, such as 3MV, Cobalt-60, or 2.5 flattening filter free (FFF), may provide a sharper penumbra, which could improve dose to OARs. This study utilizes a single patient's data who were treated initially for another benign condition; therefore, the authors acknowledge that variations in anatomy may exist across populations, which could significantly affect the feasibility of delivering treatment within acceptable constraints. While the NAc size may vary in the magnitude of millimeters between patients and with alterations with aging, it does not appear that there is a significant difference in NAc dimensions between sexes or sides (right vs left) [18]. Looking forward, it would be prudent to first observe the effects of SRS on the NAc in a large animal model. Prior studies, which ablated the NAc with stereotactic ablative surgery, observed side effects that included anhedonia and psychomotor slowing, which can significantly impact a patient’s life [10,15]. There also remains a critical ethical issue of studying vulnerable populations, such as those with significant mental health conditions like addiction [19]. Future prospective studies on human patients would need to select willing participants capable of providing informed consent. In the surgical ablation study mentioned above, the ethical recruitment and treatment of these patients were found to be compromised, with numerous patients treated without proper consent, leading to the closure of the study. Ideally, these would be patients in the "action" phase of addiction recovery with motivation to act on their condition and with strong social support coupled with a multidisciplinary team of care providers [20]. While a non-invasive modality, we caution against foregoing a patient's rights for what would be considered more socially desirable. The effects of these treatments could be debilitating and lasting and would be a serious injustice to patients if they were not fully involved and informed in their care. Additionally, the efficacy and side-effect profile of radiation in this setting would be best compared to other non-invasive neuromodulation techniques, which are increasingly being studied such as low intensity focused ultrasound (LIFU) [21].

## Conclusions

In this dosimetric feasibility study utilizing a single patient's dataset, we demonstrated the rational and technical feasibility of targeting the NAc with SRS for the management of treatment-refractory addiction. Two- and four-shot models appear to be most feasible to achieve the highest point-dose while respecting critical structure constraints. The attempt to add the six-shot will drive the optical chiasm dose over our institutional constraint of 10Gy in a single fraction due to the proximity of the NAc to the optical chiasm, making it untenable. Demonstrating the efficacy and safety of radiosurgery to the NAc for addiction will require further, carefully defined pre-clinical and clinical studies.
